# Proteinuria and the elderly: another important needle in the haystack

**DOI:** 10.1590/2175-8239-JBN-2023-E006en

**Published:** 2023-03-24

**Authors:** Marcus Gomes Bastos, Natália Maria da Silva Fernandes

**Affiliations:** 1 Universidade Federal de Juiz de Fora, Juiz de Fora, MG, Brazil.; 2 Faculdade de Ciências Médicas e da Saúde de Juiz de Fora, Juiz de Fora, MG, Brazil.; 3 Centro Universitário Governador Ozanam Coelho, Faculdade de Medicina, Ubá, MG, Brazil.

The definition of chronic kidney disease (CKD) based on functional impairment (glomerular filtration rate < 60 mL/min/1.73 m^2^), morphological changes (e.g., albuminuria), and chronicity (morphological and functional alterations present for three months or longer) proposed by the Kidney Disease Outcomes Quality Initiative (KDOQI)^1^ and subsequently referenced by the Kidney Disease Improve Global Outcomes (KDIGO)^2^ evinced the importance of CKD as a relevant public health concern associated with high morbidity and mortality^3^. CKD is a progressive condition that may affect individuals of all ages, although it is particularly prevalent among the elderly. Aging is a risk factor for several comorbidities, and in the kidney it has been associated with decreased glomerular filtration and renal parenchymal changes resulting from cellular senescence and the cumulative effect of nephrotoxic drugs prescribed to patients^4^. Thus, screening and identifying CKD in elderly individuals is particularly important to optimize health care in this important segment of the population.

In the study “A chronic kidney disease prevention campaign: proteinuria and the elderly”, Nunes et al.^5^ observed the role of proteinuria as a marker of kidney damage in elderly persons without a history of hypertension or cardiovascular disease. Few studies in medical literature have looked into this subject^6, 7, 8^. However, since elderly are at risk of CKD, screening this population for the condition is warranted^1^. This fact does not make the study less important but diminishes its originality. However, the relevance of the study by Nunes et al.^5^ has been reinforced by a recent publication highlighting that not assessing albuminuria is a missed chance to provide better kidney care to American adults9.

Another noteworthy fact is that unlike what is recommended by KDIGO 2022^2^, in the study by Nunes et al. the diagnosis of CKD was based on only one renal morphofunctional assessment. This procedure presents a methodological issue frequently found in screening studies, with consequences seldom discussed in the literature. Most of the results published in the study were within expectations, with proteinuria associated with higher blood pressure and blood glucose levels. Interestingly, age was associated with proteinuria, regardless of comorbidity, although the authors mentioned in the discussion section that when the model was adjusted for diabetes mellitus, age was no longer a risk factor for proteinuria. The discussion of proteinuria in kidney disease progression and as a marker of endothelial injury is present in the study and agrees with pertinent literature. In conclusion, the study by Nunes et al.^5^ confirmed the need to look at proteinuria in elderly patients to identify kidney disease, improve patient survival and quality of life, and decrease the cost of care in an important portion of the population.
